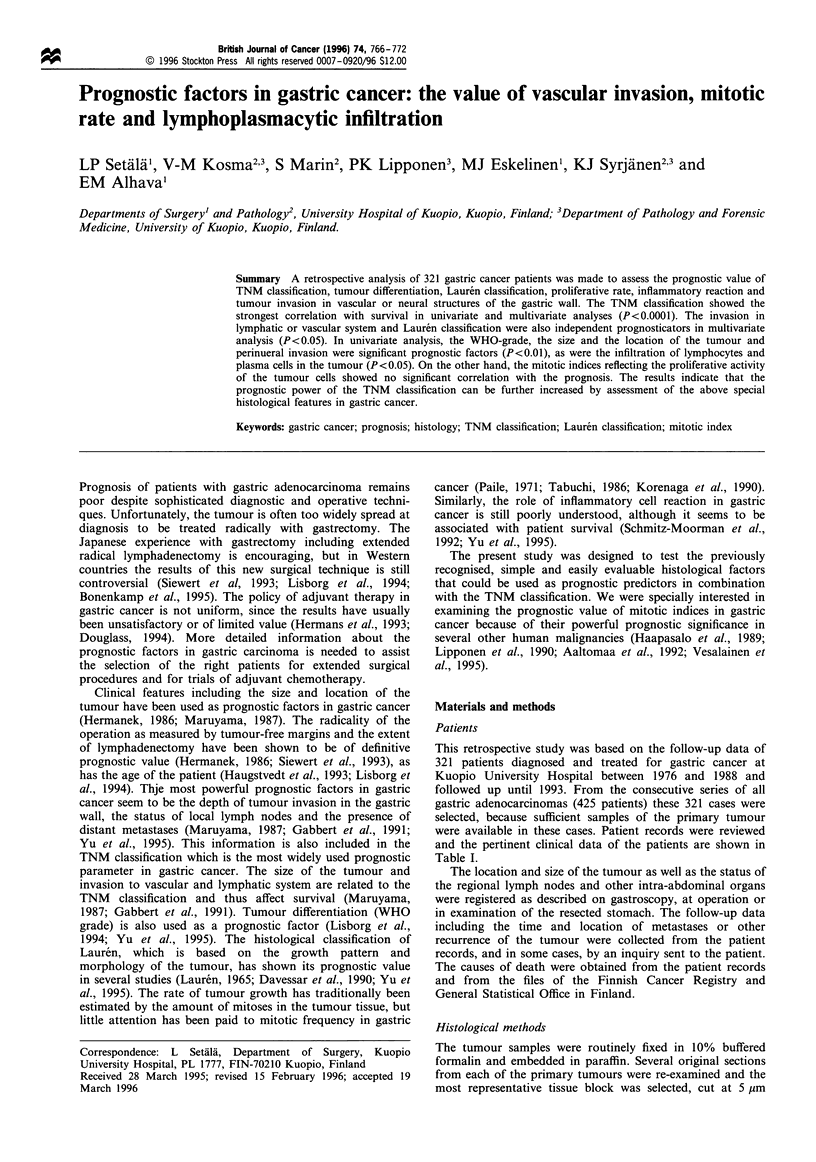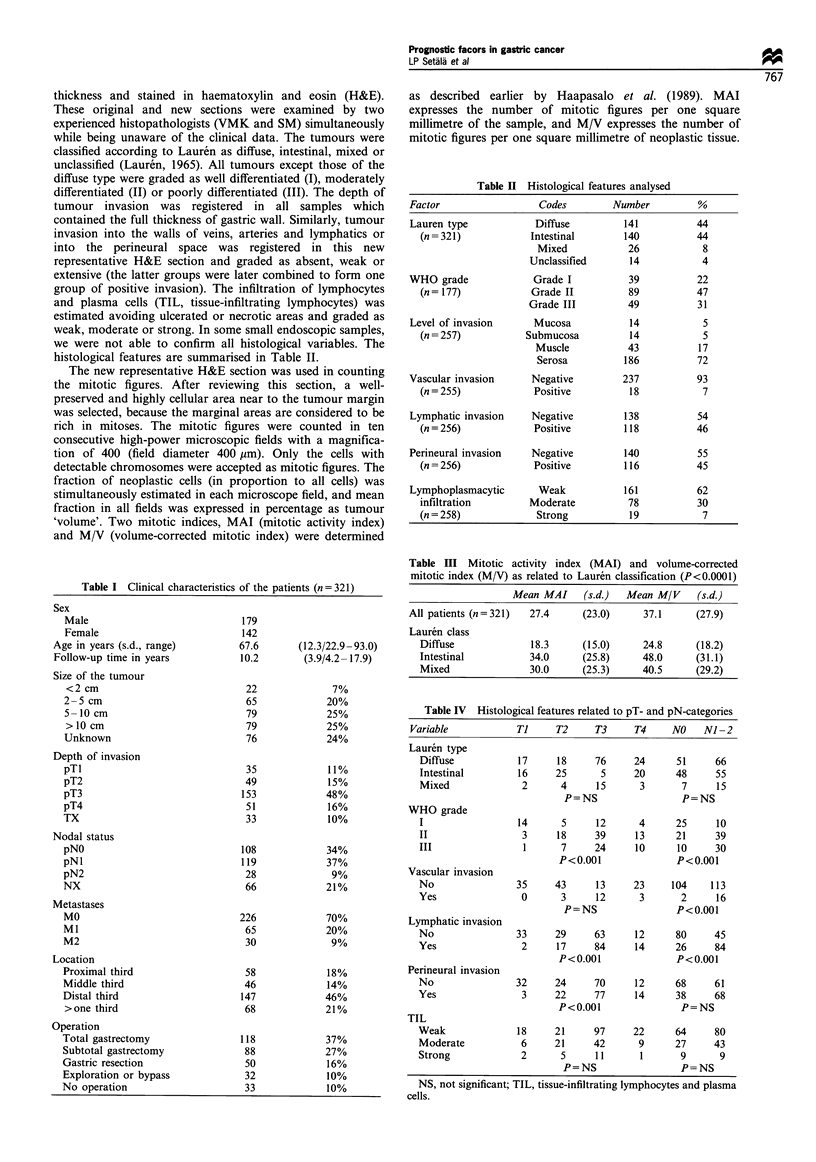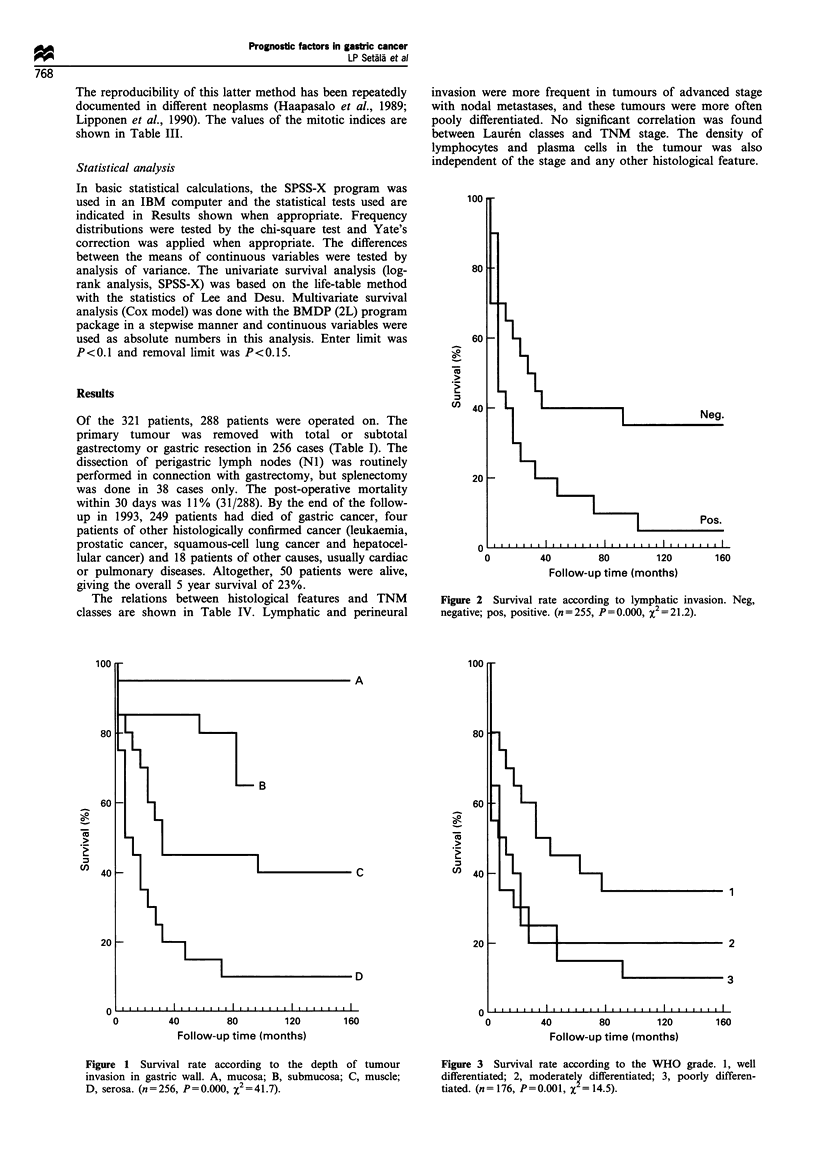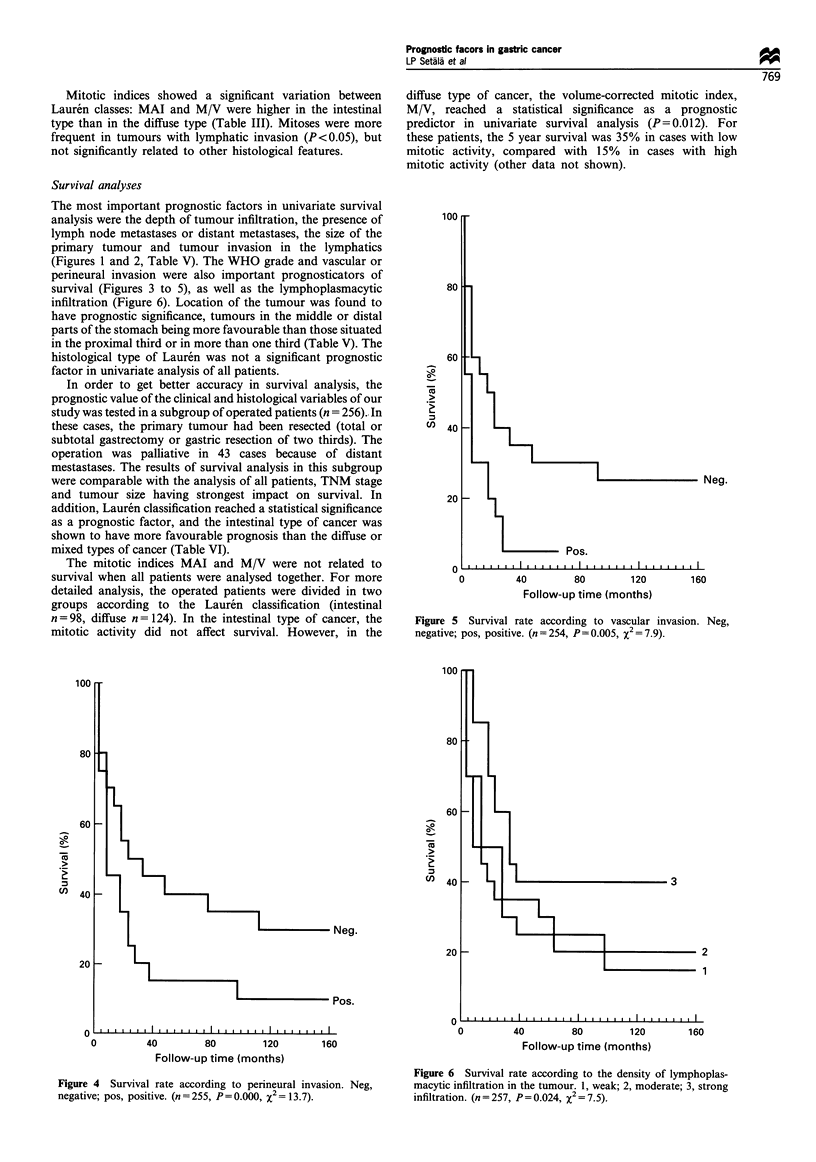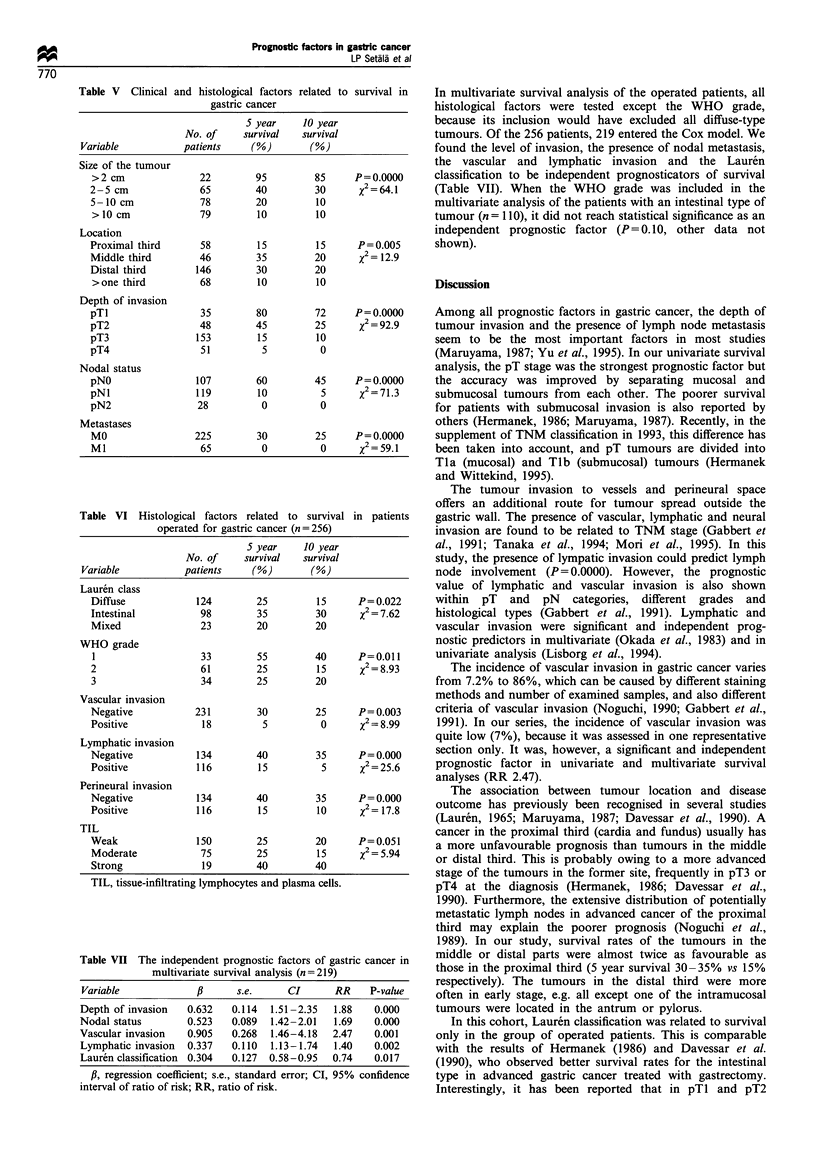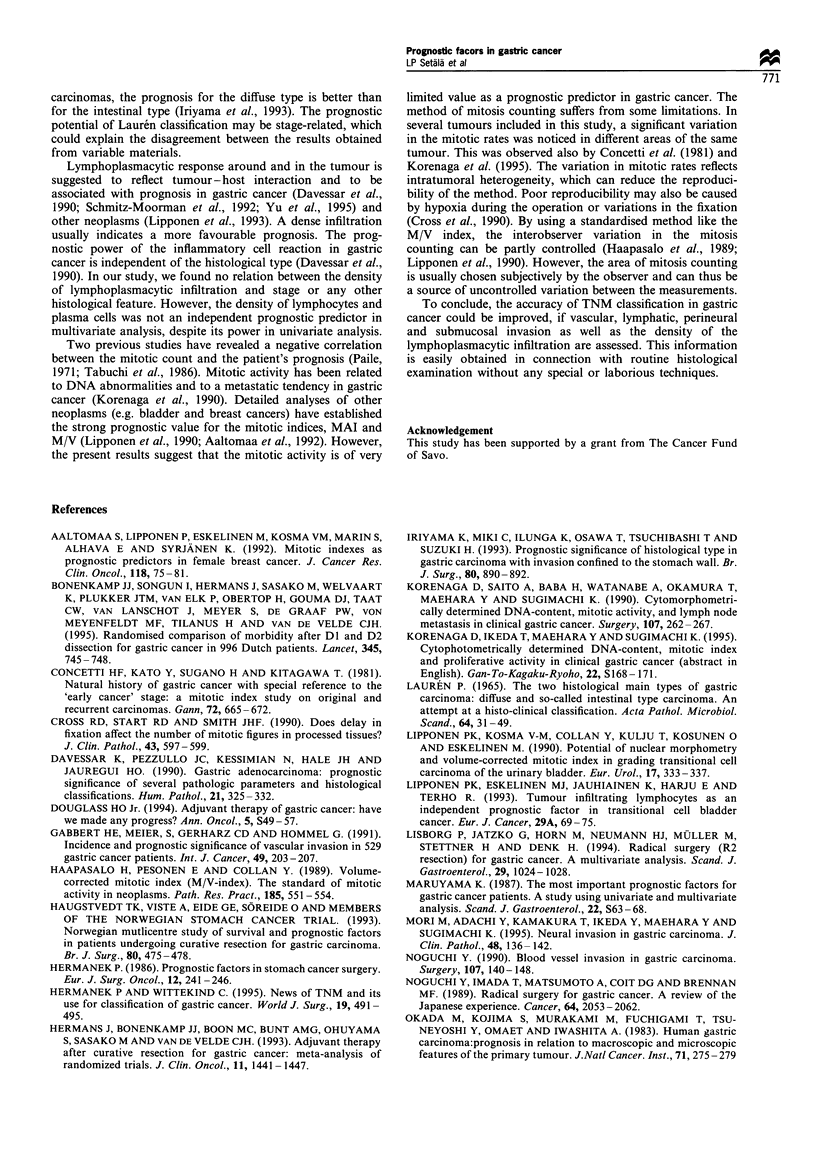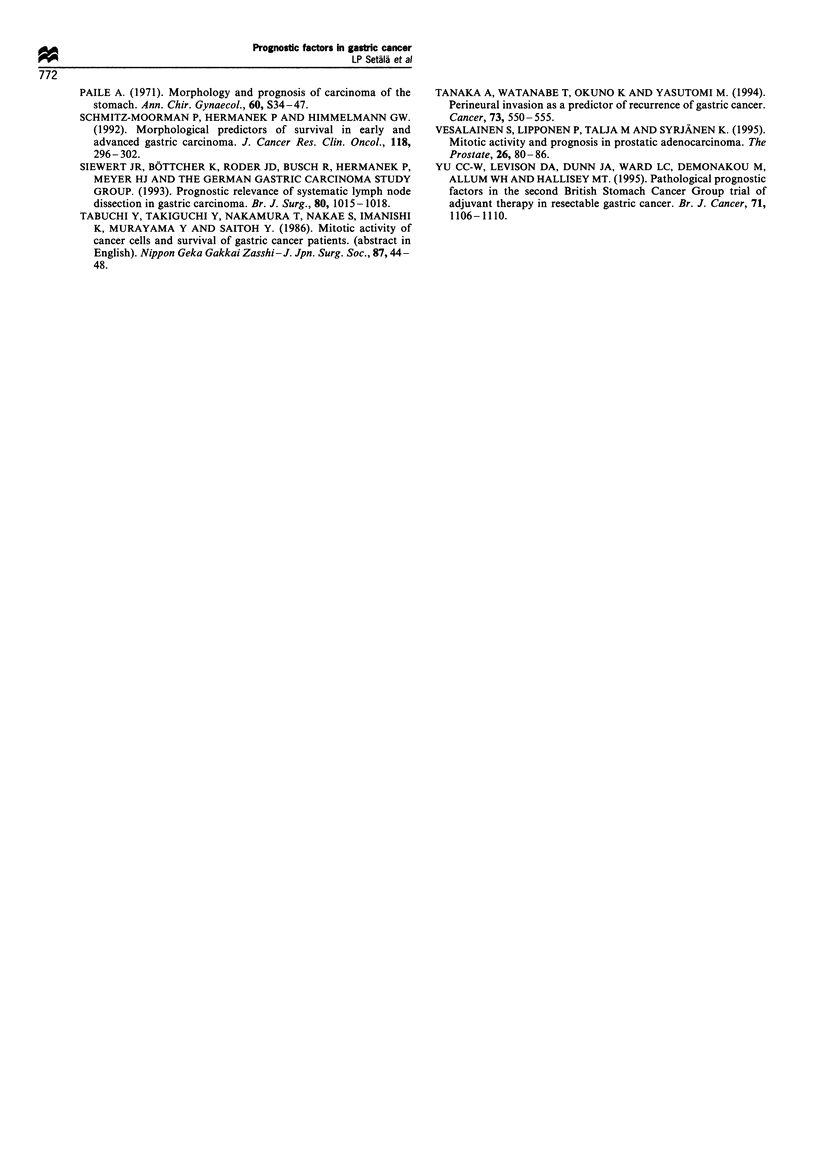# Prognostic factors in gastric cancer: the value of vascular invasion, mitotic rate and lymphoplasmacytic infiltration.

**DOI:** 10.1038/bjc.1996.434

**Published:** 1996-09

**Authors:** L. P. Setälä, V. M. Kosma, S. Marin, P. K. Lipponen, M. J. Eskelinen, K. J. Syrjänen, E. M. Alhava

**Affiliations:** Department of Surgery, University Hospital of Kuopio, Finland.

## Abstract

A retrospective analysis of 321 gastric cancer patients was made to assess the prognostic value of TNM classification, tumour differentiation, Laurén classification, proliferative rate, inflammatory reaction and tumour invasion in vascular or neural structures of the gastric wall. The TNM classification showed the strongest correlation with survival in univariate and multivariate analyses (P < 0.0001). The invasion in lymphatic or vascular system and Laurén classification were also independent prognosticators in multivariate analysis (P < 0.05). In univariate analysis, the WHO-grade, the size and the location of the tumour and perinueral invasion were significant prognostic factors (P < 0.01), as were the infiltration of lymphocytes and plasma cells in the tumour (P < 0.05). On the other hand, the mitotic indices reflecting the proliferative activity of the tumour cells showed no significant correlation with the prognosis. The results indicate that the prognostic power of the TNM classification can be further increased by assessment of the above special histological features in gastric cancer.


British Journal of Cancer (1996) 74, 766-772
© 1996 Stockton Press All rights reserved 0007-0920/96 $12.00

### Prognostic factors in gastric cancer: the value of vascular invasion, mitotic rate and lymphoplasmacytic infiltration

LP Setälä[1], V-M Kosma[2,3], S Marin[2], PK Lipponen[3], MJ Eskelinen[1], KJ Syrjänen[2,3] and EM Alhava[1]

*Departments of Surgery[1] and Pathology[2], University Hospital of Kuopio, Kuopio, Finland; [3]Department of Pathology and Forensic Medicine, University of Kuopio, Kuopio, Finland.*

**Summary** A retrospective analysis of 321 gastric cancer patients was made to assess the prognostic value of TNM classification, tumour differentiation, Laurén classification, proliferative rate, inflammatory reaction and tumour invasion in vascular or neural structures of the gastric wall. The TNM classification showed the strongest correlation with survival in univariate and multivariate analyses ($P<0.0001$). The invasion in lymphatic or vascular system and Laurén classification were also independent prognosticators in multivariate analysis ($P<0.05$). In univariate analysis, the WHO-grade, the size and the location of the tumour and perinueral invasion were significant prognostic factors ($P<0.01$), as were the infiltration of lymphocytes and plasma cells in the tumour ($P<0.05$). On the other hand, the mitotic indices reflecting the proliferative activity of the tumour cells showed no significant correlation with the prognosis. The results indicate that the prognostic power of the TNM classification can be further increased by assessment of the above special histological features in gastric cancer.

**Keywords:** gastric cancer; prognosis; histology; TNM classification; Laurén classification; mitotic index

Prognosis of patients with gastric adenocarcinoma remains poor despite sophisticated diagnostic and operative techniques. Unfortunately, the tumour is often too widely spread at diagnosis to be treated radically with gastrectomy. The Japanese experience with gastrectomy including extended radical lymphadenectomy is encouraging, but in Western countries the results of this new surgical technique is still controversial (Siewert *et al*, 1993; Lisborg *et al.*, 1994; Bonenkamp *et al.*, 1995). The policy of adjuvant therapy in gastric cancer is not uniform, since the results have usually been unsatisfactory or of limited value (Hermans *et al.*, 1993; Douglass, 1994). More detailed information about the prognostic factors in gastric carcinoma is needed to assist the selection of the right patients for extended surgical procedures and for trials of adjuvant chemotherapy.

Clinical features including the size and location of the tumour have been used as prognostic factors in gastric cancer (Hermanek, 1986; Maruyama, 1987). The radicality of the operation as measured by tumour-free margins and the extent of lymphadenectomy have been shown to be of definitive prognostic value (Hermanek, 1986; Siewert *et al.*, 1993), as has the age of the patient (Haugstvedt *et al.*, 1993; Lisborg *et al.*, 1994). Thje most powerful prognostic factors in gastric cancer seem to be the depth of tumour invasion in the gastric wall, the status of local lymph nodes and the presence of distant metastases (Maruyama, 1987; Gabbert *et al.*, 1991; Yu *et al.*, 1995). This information is also included in the TNM classification which is the most widely used prognostic parameter in gastric cancer. The size of the tumour and invasion to vascular and lymphatic system are related to the TNM classification and thus affect survival (Maruyama, 1987; Gabbert *et al.*, 1991). Tumour differentiation (WHO grade) is also used as a prognostic factor (Lisborg *et al.*, 1994; Yu *et al.*, 1995). The histological classification of Laurén, which is based on the growth pattern and morphology of the tumour, has shown its prognostic value in several studies (Laurén, 1965; Davessar *et al.*, 1990; Yu *et al.*, 1995). The rate of tumour growth has traditionally been estimated by the amount of mitoses in the tumour tissue, but little attention has been paid to mitotic frequency in gastric cancer (Paile, 1971; Tabuchi, 1986; Korenaga *et al.*, 1990). Similarly, the role of inflammatory cell reaction in gastric cancer is still poorly understood, although it seems to be associated with patient survival (Schmitz-Moorman *et al.*, 1992; Yu *et al.*, 1995).

The present study was designed to test the previously recognised, simple and easily evaluable histological factors that could be used as prognostic predictors in combination with the TNM classification. We were specially interested in examining the prognostic value of mitotic indices in gastric cancer because of their powerful prognostic significance in several other human malignancies (Haapasalo *et al.*, 1989; Lipponen *et al.*, 1990; Aaltomaa *et al.*, 1992; Vesalainen *et al.*, 1995).

### Materials and methods

### *Patients*

This retrospective study was based on the follow-up data of 321 patients diagnosed and treated for gastric cancer at Kuopio University Hospital between 1976 and 1988 and followed up until 1993. From the consecutive series of all gastric adenocarcinomas (425 patients) these 321 cases were selected, because sufficient samples of the primary tumour were available in these cases. Patient records were reviewed and the pertinent clinical data of the patients are shown in Table I.

The location and size of the tumour as well as the status of the regional lymph nodes and other intra-abdominal organs were registered as described on gastroscopy, at operation or in examination of the resected stomach. The follow-up data including the time and location of metastases or other recurrence of the tumour were collected from the patient records, and in some cases, by an inquiry sent to the patient. The causes of death were obtained from the patient records and from the files of the Finnish Cancer Registry and General Statistical Office in Finland.

### *Histological methods*

The tumour samples were routinely fixed in 10% buffered formalin and embedded in paraffin. Several original sections from each of the primary tumours were re-examined and the most representative tissue block was selected, cut at 5 $\mu m$

Correspondence: L Setälä, Department of Surgery, Kuopio University Hospital, PL 1777, FIN-70210 Kuopio, Finland
Received 28 March 1995; revised 15 February 1996; accepted 19 March 1996

thickness and stained in haematoxylin and eosin (H&E). These original and new sections were examined by two experienced histopathologists (VMK and SM) simultaneously while being unaware of the clinical data. The tumours were classified according to Laurén as diffuse, intestinal, mixed or unclassified (Laurén, 1965). All tumours except those of the diffuse type were graded as well differentiated (I), moderately differentiated (II) or poorly differentiated (III). The depth of tumour invasion was registered in all samples which contained the full thickness of gastric wall. Similarly, tumour invasion into the walls of veins, arteries and lymphatics or into the perineural space was registered in this new representative H&E section and graded as absent, weak or extensive (the latter groups were later combined to form one group of positive invasion). The infiltration of lymphocytes and plasma cells (TIL, tissue-infiltrating lymphocytes) was estimated avoiding ulcerated or necrotic areas and graded as weak, moderate or strong. In some small endoscopic samples, we were not able to confirm all histological variables. The histological features are summarised in Table II.

The new representative H&E section was used in counting the mitotic figures. After reviewing this section, a well-preserved and highly cellular area near to the tumour margin was selected, because the marginal areas are considered to be rich in mitoses. The mitotic figures were counted in ten consecutive high-power microscopic fields with a magnification of 400 (field diameter 400 μm). Only the cells with detectable chromosomes were accepted as mitotic figures. The fraction of neoplastic cells (in proportion to all cells) was stimultaneously estimated in each microscope field, and mean fraction in all fields was expressed in percentage as tumour 'volume'. Two mitotic indices, MAI (mitotic activity index) and M/V (volume-corrected mitotic index) were determined as described earlier by Haapasalo *et al.* (1989). MAI expresses the number of mitotic figures per one square millimetre of the sample, and M/V expresses the number of mitotic figures per one square millimetre of neoplastic tissue.

**Table I** Clinical characteristics of the patients ($n=321$)

| | | |
|---|---|---|
| Sex | | |
| Male | 179 | |
| Female | 142 | |
| Age in years (s.d., range) | 67.6 | (12.3/22.9–93.0) |
| Follow-up time in years | 10.2 | (3.9/4.2–17.9) |
| Size of the tumour | | |
| <2 cm | 22 | 7% |
| 2–5 cm | 65 | 20% |
| 5–10 cm | 79 | 25% |
| >10 cm | 79 | 25% |
| Unknown | 76 | 24% |
| Depth of invasion | | |
| pT1 | 35 | 11% |
| pT2 | 49 | 15% |
| pT3 | 153 | 48% |
| pT4 | 51 | 16% |
| TX | 33 | 10% |
| Nodal status | | |
| pN0 | 108 | 34% |
| pN1 | 119 | 37% |
| pN2 | 28 | 9% |
| NX | 66 | 21% |
| Metastases | | |
| M0 | 226 | 70% |
| M1 | 65 | 20% |
| M2 | 30 | 9% |
| Location | | |
| Proximal third | 58 | 18% |
| Middle third | 46 | 14% |
| Distal third | 147 | 46% |
| >one third | 68 | 21% |
| Operation | | |
| Total gastrectomy | 118 | 37% |
| Subtotal gastrectomy | 88 | 27% |
| Gastric resection | 50 | 16% |
| Exploration or bypass | 32 | 10% |
| No operation | 33 | 10% |

**Table II** Histological features analysed

| *Factor* | *Codes* | *Number* | *%* |
|---|---|---|---|
| Lauren type ($n=321$) | Diffuse | 141 | 44 |
| | Intestinal | 140 | 44 |
| | Mixed | 26 | 8 |
| | Unclassified | 14 | 4 |
| WHO grade ($n=177$) | Grade I | 39 | 22 |
| | Grade II | 89 | 47 |
| | Grade III | 49 | 31 |
| Level of invasion ($n=257$) | Mucosa | 14 | 5 |
| | Submucosa | 14 | 5 |
| | Muscle | 43 | 17 |
| | Serosa | 186 | 72 |
| Vascular invasion ($n=255$) | Negative | 237 | 93 |
| | Positive | 18 | 7 |
| Lymphatic invasion ($n=256$) | Negative | 138 | 54 |
| | Positive | 118 | 46 |
| Perineural invasion ($n=256$) | Negative | 140 | 55 |
| | Positive | 116 | 45 |
| Lymphoplasmacytic infiltration ($n=258$) | Weak | 161 | 62 |
| | Moderate | 78 | 30 |
| | Strong | 19 | 7 |

**Table III** Mitotic activity index (MAI) and volume-corrected mitotic index (M/V) as related to Laurén classification ($P<0.0001$)

| | *Mean MAI* | *(s.d.)* | *Mean M/V* | *(s.d.)* |
|---|---|---|---|---|
| All patients ($n=321$) | 27.4 | (23.0) | 37.1 | (27.9) |
| Laurén class | | | | |
| Diffuse | 18.3 | (15.0) | 24.8 | (18.2) |
| Intestinal | 34.0 | (25.8) | 48.0 | (31.1) |
| Mixed | 30.0 | (25.3) | 40.5 | (29.2) |

**Table IV** Histological features related to pT- and pN-categories

| *Variable* | *T1* | *T2* | *T3* | *T4* | *N0* | *N1–2* |
|---|---|---|---|---|---|---|
| Laurén type | | | | | | |
| Diffuse | 17 | 18 | 76 | 24 | 51 | 66 |
| Intestinal | 16 | 25 | 5 | 20 | 48 | 55 |
| Mixed | 2 | 4 | 15 | 3 | 7 | 15 |
| | | $P=$NS | | | $P=$NS | |
| WHO grade | | | | | | |
| I | 14 | 5 | 12 | 4 | 25 | 10 |
| II | 3 | 18 | 39 | 13 | 21 | 39 |
| III | 1 | 7 | 24 | 10 | 10 | 30 |
| | | $P<0.001$ | | | $P<0.001$ | |
| Vascular invasion | | | | | | |
| No | 35 | 43 | 13 | 23 | 104 | 113 |
| Yes | 0 | 3 | 12 | 3 | 2 | 16 |
| | | $P=$NS | | | $P<0.001$ | |
| Lymphatic invasion | | | | | | |
| No | 33 | 29 | 63 | 12 | 80 | 45 |
| Yes | 2 | 17 | 84 | 14 | 26 | 84 |
| | | $P<0.001$ | | | $P<0.001$ | |
| Perineural invasion | | | | | | |
| No | 32 | 24 | 70 | 12 | 68 | 61 |
| Yes | 3 | 22 | 77 | 14 | 38 | 68 |
| | | $P<0.001$ | | | $P=$NS | |
| TIL | | | | | | |
| Weak | 18 | 21 | 97 | 22 | 64 | 80 |
| Moderate | 6 | 21 | 42 | 9 | 27 | 43 |
| Strong | 2 | 5 | 11 | 1 | 9 | 9 |
| | | $P=$NS | | | $P=$NS | |

NS, not significant; TIL, tissue-infiltrating lymphocytes and plasma cells.

The reproducibility of this latter method has been repeatedly documented in different neoplasms (Haapasalo *et al.*, 1989; Lipponen *et al.*, 1990). The values of the mitotic indices are shown in Table III.

*Statistical analysis*

In basic statistical calculations, the SPSS-X program was used in an IBM computer and the statistical tests used are indicated in Results shown when appropriate. Frequency distributions were tested by the chi-square test and Yate's correction was applied when appropriate. The differences between the means of continuous variables were tested by analysis of variance. The univariate survival analysis (log-rank analysis, SPSS-X) was based on the life-table method with the statistics of Lee and Desu. Multivariate survival analysis (Cox model) was done with the BMDP (2L) program package in a stepwise manner and continuous variables were used as absolute numbers in this analysis. Enter limit was $P<0.1$ and removal limit was $P<0.15$.

### Results

Of the 321 patients, 288 patients were operated on. The primary tumour was removed with total or subtotal gastrectomy or gastric resection in 256 cases (Table I). The dissection of perigastric lymph nodes (N1) was routinely performed in connection with gastrectomy, but splenectomy was done in 38 cases only. The post-operative mortality within 30 days was 11% (31/288). By the end of the follow-up in 1993, 249 patients had died of gastric cancer, four patients of other histologically confirmed cancer (leukaemia, prostatic cancer, squamous-cell lung cancer and hepatocellular cancer) and 18 patients of other causes, usually cardiac or pulmonary diseases. Altogether, 50 patients were alive, giving the overall 5 year survival of 23%.

The relations between histological features and TNM classes are shown in Table IV. Lymphatic and perineural invasion were more frequent in tumours of advanced stage with nodal metastases, and these tumours were more often pooly differentiated. No significant correlation was found between Laurén classes and TNM stage. The density of lymphocytes and plasma cells in the tumour was also independent of the stage and any other histological feature.

**Figure 2** Survival rate according to lymphatic invasion. Neg, negative; pos, positive. ($n=255$, $P=0.000$, $\chi^2=21.2$).

**Figure 1** Survival rate according to the depth of tumour invasion in gastric wall. A, mucosa; B, submucosa; C, muscle; D, serosa. ($n=256$, $P=0.000$, $\chi^2=41.7$).

**Figure 3** Survival rate according to the WHO grade. 1, well differentiated; 2, moderately differentiated; 3, poorly differentiated. ($n=176$, $P=0.001$, $\chi^2=14.5$).

Mitotic indices showed a significant variation between Laurén classes: MAI and M/V were higher in the intestinal type than in the diffuse type (Table III). Mitoses were more frequent in tumours with lymphatic invasion ($P<0.05$), but not significantly related to other histological features.

*Survival analyses*

The most important prognostic factors in univariate survival analysis were the depth of tumour infiltration, the presence of lymph node metastases or distant metastases, the size of the primary tumour and tumour invasion in the lymphatics (Figures 1 and 2, Table V). The WHO grade and vascular or perineural invasion were also important prognosticators of survival (Figures 3 to 5), as well as the lymphoplasmacytic infiltration (Figure 6). Location of the tumour was found to have prognostic significance, tumours in the middle or distal parts of the stomach being more favourable than those situated in the proximal third or in more than one third (Table V). The histological type of Laurén was not a significant prognostic factor in univariate analysis of all patients.

In order to get better accuracy in survival analysis, the prognostic value of the clinical and histological variables of our study was tested in a subgroup of operated patients ($n=256$). In these cases, the primary tumour had been resected (total or subtotal gastrectomy or gastric resection of two thirds). The operation was palliative in 43 cases because of distant mestastases. The results of survival analysis in this subgroup were comparable with the analysis of all patients, TNM stage and tumour size having strongest impact on survival. In addition, Laurén classification reached a statistical significance as a prognostic factor, and the intestinal type of cancer was shown to have more favourable prognosis than the diffuse or mixed types of cancer (Table VI).

The mitotic indices MAI and M/V were not related to survival when all patients were analysed together. For more detailed analysis, the operated patients were divided in two groups according to the Laurén classification (intestinal $n=98$, diffuse $n=124$). In the intestinal type of cancer, the mitotic activity did not affect survival. However, in the diffuse type of cancer, the volume-corrected mitotic index, M/V, reached a statistical significance as a prognostic predictor in univariate survival analysis ($P=0.012$). For these patients, the 5 year survival was 35% in cases with low mitotic activity, compared with 15% in cases with high mitotic activity (other data not shown).

**Figure 5** Survival rate according to vascular invasion. Neg, negative; pos, positive. ($n=254$, $P=0.005$, $\chi^2=7.9$).

**Figure 4** Survival rate according to perineural invasion. Neg, negative; pos, positive. ($n=255$, $P=0.000$, $\chi^2=13.7$).

**Figure 6** Survival rate according to the density of lymphoplasmacytic infiltration in the tumour. 1, weak; 2, moderate; 3, strong infiltration. ($n=257$, $P=0.024$, $\chi^2=7.5$).

**Table V** Clinical and histological factors related to survival in gastric cancer

| Variable | No. of patients | 5 year survival (%) | 10 year survival (%) | |
|---|---|---|---|---|
| Size of the tumour | | | | |
| >2 cm | 22 | 95 | 85 | $P=0.0000$ |
| 2–5 cm | 65 | 40 | 30 | $\chi^2=64.1$ |
| 5–10 cm | 78 | 20 | 10 | |
| >10 cm | 79 | 10 | 10 | |
| Location | | | | |
| Proximal third | 58 | 15 | 15 | $P=0.005$ |
| Middle third | 46 | 35 | 20 | $\chi^2=12.9$ |
| Distal third | 146 | 30 | 20 | |
| >one third | 68 | 10 | 10 | |
| Depth of invasion | | | | |
| pT1 | 35 | 80 | 72 | $P=0.0000$ |
| pT2 | 48 | 45 | 25 | $\chi^2=92.9$ |
| pT3 | 153 | 15 | 10 | |
| pT4 | 51 | 5 | 0 | |
| Nodal status | | | | |
| pN0 | 107 | 60 | 45 | $P=0.0000$ |
| pN1 | 119 | 10 | 5 | $\chi^2=71.3$ |
| pN2 | 28 | 0 | 0 | |
| Metastases | | | | |
| M0 | 225 | 30 | 25 | $P=0.0000$ |
| M1 | 65 | 0 | 0 | $\chi^2=59.1$ |

**Table VI** Histological factors related to survival in patients operated for gastric cancer ($n=256$)

| Variable | No. of patients | 5 year survival (%) | 10 year survival (%) | |
|---|---|---|---|---|
| Laurén class | | | | |
| Diffuse | 124 | 25 | 15 | $P=0.022$ |
| Intestinal | 98 | 35 | 30 | $\chi^2=7.62$ |
| Mixed | 23 | 20 | 20 | |
| WHO grade | | | | |
| 1 | 33 | 55 | 40 | $P=0.011$ |
| 2 | 61 | 25 | 15 | $\chi^2=8.93$ |
| 3 | 34 | 25 | 20 | |
| Vascular invasion | | | | |
| Negative | 231 | 30 | 25 | $P=0.003$ |
| Positive | 18 | 5 | 0 | $\chi^2=8.99$ |
| Lymphatic invasion | | | | |
| Negative | 134 | 40 | 35 | $P=0.000$ |
| Positive | 116 | 15 | 5 | $\chi^2=25.6$ |
| Perineural invasion | | | | |
| Negative | 134 | 40 | 35 | $P=0.000$ |
| Positive | 116 | 15 | 10 | $\chi^2=17.8$ |
| TIL | | | | |
| Weak | 150 | 25 | 20 | $P=0.051$ |
| Moderate | 75 | 25 | 15 | $\chi^2=5.94$ |
| Strong | 19 | 40 | 40 | |

TIL, tissue-infiltrating lymphocytes and plasma cells.

**Table VII** The independent prognostic factors of gastric cancer in multivariate survival analysis ($n=219$)

| Variable | $\beta$ | s.e. | CI | RR | P-value |
|---|---|---|---|---|---|
| Depth of invasion | 0.632 | 0.114 | 1.51–2.35 | 1.88 | 0.000 |
| Nodal status | 0.523 | 0.089 | 1.42–2.01 | 1.69 | 0.000 |
| Vascular invasion | 0.905 | 0.268 | 1.46–4.18 | 2.47 | 0.001 |
| Lymphatic invasion | 0.337 | 0.110 | 1.13–1.74 | 1.40 | 0.002 |
| Laurén classification | 0.304 | 0.127 | 0.58–0.95 | 0.74 | 0.017 |

$\beta$, regression coefficient; s.e., standard error; CI, 95% confidence interval of ratio of risk; RR, ratio of risk.

In multivariate survival analysis of the operated patients, all histological factors were tested except the WHO grade, because its inclusion would have excluded all diffuse-type tumours. Of the 256 patients, 219 entered the Cox model. We found the level of invasion, the presence of nodal metastasis, the vascular and lymphatic invasion and the Laurén classification to be independent prognosticators of survival (Table VII). When the WHO grade was included in the multivariate analysis of the patients with an intestinal type of tumour ($n=110$), it did not reach statistical significance as an independent prognostic factor ($P=0.10$, other data not shown).

### Discussion

Among all prognostic factors in gastric cancer, the depth of tumour invasion and the presence of lymph node metastasis seem to be the most important factors in most studies (Maruyama, 1987; Yu *et al.*, 1995). In our univariate survival analysis, the pT stage was the strongest prognostic factor but the accuracy was improved by separating mucosal and submucosal tumours from each other. The poorer survival for patients with submucosal invasion is also reported by others (Hermanek, 1986; Maruyama, 1987). Recently, in the supplement of TNM classification in 1993, this difference has been taken into account, and pT tumours are divided into T1a (mucosal) and T1b (submucosal) tumours (Hermanek and Wittekind, 1995).

The tumour invasion to vessels and perineural space offers an additional route for tumour spread outside the gastric wall. The presence of vascular, lymphatic and neural invasion are found to be related to TNM stage (Gabbert *et al.*, 1991; Tanaka *et al.*, 1994; Mori *et al.*, 1995). In this study, the presence of lympatic invasion could predict lymph node involvement ($P=0.0000$). However, the prognostic value of lymphatic and vascular invasion is also shown within pT and pN categories, different grades and histological types (Gabbert *et al.*, 1991). Lymphatic and vascular invasion were significant and independent prognostic predictors in multivariate (Okada *et al.*, 1983) and in univariate analysis (Lisborg *et al.*, 1994).

The incidence of vascular invasion in gastric cancer varies from 7.2% to 86%, which can be caused by different staining methods and number of examined samples, and also different criteria of vascular invasion (Noguchi, 1990; Gabbert *et al.*, 1991). In our series, the incidence of vascular invasion was quite low (7%), because it was assessed in one representative section only. It was, however, a significant and independent prognostic factor in univariate and multivariate survival analyses (RR 2.47).

The association between tumour location and disease outcome has previously been recognised in several studies (Laurén, 1965; Maruyama, 1987; Davessar *et al.*, 1990). A cancer in the proximal third (cardia and fundus) usually has a more unfavourable prognosis than tumours in the middle or distal third. This is probably owing to a more advanced stage of the tumours in the former site, frequently in pT3 or pT4 at the diagnosis (Hermanek, 1986; Davessar *et al.*, 1990). Furthermore, the extensive distribution of potentially metastatic lymph nodes in advanced cancer of the proximal third may explain the poorer prognosis (Noguchi *et al.*, 1989). In our study, survival rates of the tumours in the middle or distal parts were almost twice as favourable as those in the proximal third (5 year survival 30–35% *vs* 15% respectively). The tumours in the distal third were more often in early stage, e.g. all except one of the intramucosal tumours were located in the antrum or pylorus.

In this cohort, Laurén classification was related to survival only in the group of operated patients. This is comparable with the results of Hermanek (1986) and Davessar *et al.* (1990), who observed better survival rates for the intestinal type in advanced gastric cancer treated with gastrectomy. Interestingly, it has been reported that in pT1 and pT2

carcinomas, the prognosis for the diffuse type is better than for the intestinal type (Iriyama *et al.*, 1993). The prognostic potential of Laurén classification may be stage-related, which could explain the disagreement between the results obtained from variable materials.

Lymphoplasmacytic response around and in the tumour is suggested to reflect tumour–host interaction and to be associated with prognosis in gastric cancer (Davessar *et al.*, 1990; Schmitz-Moorman *et al.*, 1992; Yu *et al.*, 1995) and other neoplasms (Lipponen *et al.*, 1993). A dense infiltration usually indicates a more favourable prognosis. The prognostic power of the inflammatory cell reaction in gastric cancer is independent of the histological type (Davessar *et al.*, 1990). In our study, we found no relation between the density of lymphoplasmacytic infiltration and stage or any other histological feature. However, the density of lymphocytes and plasma cells was not an independent prognostic predictor in multivariate analysis, despite its power in univariate analysis.

Two previous studies have revealed a negative correlation between the mitotic count and the patient's prognosis (Paile, 1971; Tabuchi *et al.*, 1986). Mitotic activity has been related to DNA abnormalities and to a metastatic tendency in gastric cancer (Korenaga *et al.*, 1990). Detailed analyses of other neoplasms (e.g. bladder and breast cancers) have established the strong prognostic value for the mitotic indices, MAI and M/V (Lipponen *et al.*, 1990; Aaltomaa *et al.*, 1992). However, the present results suggest that the mitotic activity is of very limited value as a prognostic predictor in gastric cancer. The method of mitosis counting suffers from some limitations. In several tumours included in this study, a significant variation in the mitotic rates was noticed in different areas of the same tumour. This was observed also by Concetti *et al.* (1981) and Korenaga *et al.* (1995). The variation in mitotic rates reflects intratumoral heterogeneity, which can reduce the reproducibility of the method. Poor reproducibility may also be caused by hypoxia during the operation or variations in the fixation (Cross *et al.*, 1990). By using a standardised method like the M/V index, the interobserver variation in the mitosis counting can be partly controlled (Haapasalo *et al.*, 1989; Lipponen *et al.*, 1990). However, the area of mitosis counting is usually chosen subjectively by the observer and can thus be a source of uncontrolled variation between the measurements.

To conclude, the accuracy of TNM classification in gastric cancer could be improved, if vascular, lymphatic, perineural and submucosal invasion as well as the density of the lymphoplasmacytic infiltration are assessed. This information is easily obtained in connection with routine histological examination without any special or laborious techniques.

**Acknowledgement**

This study has been supported by a grant from The Cancer Fund of Savo.

### References

AALTOMAA S, LIPPONEN P, ESKELINEN M, KOSMA VM, MARIN S, ALHAVA E AND SYRJÄNEN K. (1992). Mitotic indexes as prognostic predictors in female breast cancer. *J. Cancer Res. Clin. Oncol.*, **118**, 75–81.

BONENKAMP JJ, SONGUN I, HERMANS J, SASAKO M, WELVAART K, PLUKKER JTM, VAN ELK P, OBERTOP H, GOUMA DJ, TAAT CW, VAN LANSCHOT J, MEYER S, DE GRAAF PW, VON MEYENFELDT MF, TILANUS H AND VAN DE VELDE CJH. (1995). Randomised comparison of morbidity after D1 and D2 dissection for gastric cancer in 996 Dutch patients. *Lancet*, **345**, 745–748.

CONCETTI HF, KATO Y, SUGANO H AND KITAGAWA T. (1981). Natural history of gastric cancer with special reference to the 'early cancer' stage: a mitotic index study on original and recurrent carcinomas. *Gann*, **72**, 665–672.

CROSS RD, START RD AND SMITH JHF. (1990). Does delay in fixation affect the number of mitotic figures in processed tissues? *J. Clin. Pathol.*, **43**, 597–599.

DAVESSAR K, PEZZULLO JC, KESSIMIAN N, HALE JH AND JAUREGUI HO. (1990). Gastric adenocarcinoma: prognostic significance of several pathologic parameters and histological classifications. *Hum. Pathol.*, **21**, 325–332.

DOUGLASS HO Jr. (1994). Adjuvant therapy of gastric cancer: have we made any progress? *Ann. Oncol.*, **5**, S49–57.

GABBERT HE, MEIER, S, GERHARZ CD AND HOMMEL G. (1991). Incidence and prognostic significance of vascular invasion in 529 gastric cancer patients. *Int. J. Cancer*, **49**, 203–207.

HAAPASALO H, PESONEN E AND COLLAN Y. (1989). Volume-corrected mitotic index (M/V-index). The standard of mitotic activity in neoplasms. *Path. Res. Pract.*, **185**, 551–554.

HAUGSTVEDT TK, VISTE A, EIDE GE, SÖREIDE O AND MEMBERS OF THE NORWEGIAN STOMACH CANCER TRIAL. (1993). Norwegian mutlicentre study of survival and prognostic factors in patients undergoing curative resection for gastric carcinoma. *Br. J. Surg.*, **80**, 475–478.

HERMANEK P. (1986). Prognostic factors in stomach cancer surgery. *Eur. J. Surg. Oncol.*, **12**, 241–246.

HERMANEK P AND WITTEKIND C. (1995). News of TNM and its use for classification of gastric cancer. *World J. Surg.*, **19**, 491–495.

HERMANS J, BONENKAMP JJ, BOON MC, BUNT AMG, OHUYAMA S, SASAKO M AND VAN DE VELDE CJH. (1993). Adjuvant therapy after curative resection for gastric cancer: meta-analysis of randomized trials. *J. Clin. Oncol.*, **11**, 1441–1447.

IRIYAMA K, MIKI C, ILUNGA K, OSAWA T, TSUCHIBASHI T AND SUZUKI H. (1993). Prognostic significance of histological type in gastric carcinoma with invasion confined to the stomach wall. *Br. J. Surg.*, **80**, 890–892.

KORENAGA D, SAITO A, BABA H, WATANABE A, OKAMURA T, MAEHARA Y AND SUGIMACHI K. (1990). Cytomorphometrically determined DNA-content, mitotic activity, and lymph node metastasis in clinical gastric cancer. *Surgery*, **107**, 262–267.

KORENAGA D, IKEDA T, MAEHARA Y AND SUGIMACHI K. (1995). Cytophotometrically determined DNA-content, mitotic index and proliferative activity in clinical gastric cancer (abstract in English). *Gan-To-Kagaku-Ryoho*, **22**, S168–171.

LAURÉN P. (1965). The two histological main types of gastric carcinoma: diffuse and so-called intestinal type carcinoma. An attempt at a histo-clinical classification. *Acta Pathol. Microbiol. Scand.*, **64**, 31–49.

LIPPONEN PK, KOSMA V-M, COLLAN Y, KULJU T, KOSUNEN O AND ESKELINEN M. (1990). Potential of nuclear morphometry and volume-corrected mitotic index in grading transitional cell carcinoma of the urinary bladder. *Eur. Urol.*, **17**, 333–337.

LIPPONEN PK, ESKELINEN MJ, JAUHIAINEN K, HARJU E AND TERHO R. (1993). Tumour infiltrating lymphocytes as an independent prognostic factor in transitional cell bladder cancer. *Eur. J. Cancer*, **29A**, 69–75.

LISBORG P, JATZKO G, HORN M, NEUMANN HJ, MÜLLER M, STETTNER H AND DENK H. (1994). Radical surgery (R2 resection) for gastric cancer. A multivariate analysis. *Scand. J. Gastroenterol.*, **29**, 1024–1028.

MARUYAMA K. (1987). The most important prognostic factors for gastric cancer patients. A study using univariate and multivariate analysis. *Scand. J. Gastroenterol.*, **22**, S63–68.

MORI M, ADACHI Y, KAMAKURA T, IKEDA Y, MAEHARA Y AND SUGIMACHI K. (1995). Neural invasion in gastric carcinoma. *J. Clin. Pathol.*, **48**, 136–142.

NOGUCHI Y. (1990). Blood vessel invasion in gastric carcinoma. *Surgery*, **107**, 140–148.

NOGUCHI Y, IMADA T, MATSUMOTO A, COIT DG AND BRENNAN MF. (1989). Radical surgery for gastric cancer. A review of the Japanese experience. *Cancer*, **64**, 2053–2062.

OKADA M, KOJIMA S, MURAKAMI M, FUCHIGAMI T, TSUNEYOSHI Y, OMAET AND IWASHITA A. (1983). Human gastric carcinoma:prognosis in relation to macroscopic and microscopic features of the primary tumour. *J.Natl Cancer. Inst.*, **71**, 275–279

PAILE A. (1971). Morphology and prognosis of carcinoma of the stomach. *Ann. Chir. Gynaecol.*, **60**, S34-47.

SCHMITZ-MOORMAN P, HERMANEK P AND HIMMELMANN GW. (1992). Morphological predictors of survival in early and advanced gastric carcinoma. *J. Cancer Res. Clin. Oncol.*, **118**, 296-302.

SIEWERT JR, BÖTTCHER K, RODER JD, BUSCH R, HERMANEK P, MEYER HJ AND THE GERMAN GASTRIC CARCINOMA STUDY GROUP. (1993). Prognostic relevance of systematic lymph node dissection in gastric carcinoma. *Br. J. Surg.*, **80**, 1015-1018.

TABUCHI Y, TAKIGUCHI Y, NAKAMURA T, NAKAE S, IMANISHI K, MURAYAMA Y AND SAITOH Y. (1986). Mitotic activity of cancer cells and survival of gastric cancer patients. (abstract in English). *Nippon Geka Gakkai Zasshi-J. Jpn. Surg. Soc.*, **87**, 44-48.

TANAKA A, WATANABE T, OKUNO K AND YASUTOMI M. (1994). Perineural invasion as a predictor of recurrence of gastric cancer. *Cancer*, **73**, 550-555.

VESALAINEN S, LIPPONEN P, TALJA M AND SYRJÄNEN K. (1995). Mitotic activity and prognosis in prostatic adenocarcinoma. *The Prostate*, **26**, 80-86.

YU CC-W, LEVISON DA, DUNN JA, WARD LC, DEMONAKOU M, ALLUM WH AND HALLISEY MT. (1995). Pathological prognostic factors in the second British Stomach Cancer Group trial of adjuvant therapy in resectable gastric cancer. *Br. J. Cancer*, **71**, 1106-1110.